# Peeking into the future: Transdermal patches for the delivery of micronutrient supplements

**DOI:** 10.1016/j.metop.2021.100109

**Published:** 2021-07-13

**Authors:** Maria G. Grammatikopoulou, Konstantinos Gkiouras, Efthimios Dardiotis, Efterpi Zafiriou, Christina Tsigalou, Dimitrios P. Bogdanos

**Affiliations:** a Department of Rheumatology and Clinical Immunology, Faculty of Medicine, School of Health Sciences, University of Thessaly, Larissa, Greece; b Department of Nutritional Sciences & Dietetics, Faculty of Health Sciences, International Hellenic University, Alexander Campus, Thessaloniki, Greece; c Laboratory of Clinical Pharmacology, Faculty of Medicine, School of Health Sciences, Aristotle University of Thessaloniki, Thessaloniki, Greece; d Department of Neurology, Faculty of Medicine, School of Health Sciences, University of Thessaly, Larissa, Greece; e Department of Dermatology, Faculty of Medicine, School of Health Sciences, University of Thessaly, Larissa, Greece; f Department of Microbiology, Democritus University of Thrace, Alexandroupolis, Greece

**Keywords:** Vitamin, Multivitamin, Dietary intake, Dietary supplements, Iron, Vitamin D, Nutrient deficiency, Hidden hunger, Micronutrient, Minerals

## Abstract

Adhesive transdermal delivery devices (patches) are the latest advancement in the delivery of micronutrients. A common challenge in this mode of delivery includes surpassing the physical barrier of the skin, while the use of microneedle (MN) arrays, or pretreatment of the skin with MNs can be used for a more successful outcome. Limited evidence from human non-randomized trials point to a sub-optimal delivery of iron through skin patches, although no MNs were used in those trials. Moreover, the use of patches proved inefficient in reducing the prevalence of micronutrient deficiencies in post-bariatric surgery patients. The delivery of minerals was tested in animals using reservoir-type patches, gel/foam patches, MNs and iontophoresis. Results from these studies indicate a possible interplay between the dietary manipulation of mineral intake and the trandermal delivery through patches, as reduced, or regular dietary intake seems to increase absorption of the delivered mineral. Moreover, intervention duration could be an additional factor affecting absorption. Possible adverse events from animal studies include redness or decolorization of skin. *In vitro* and *ex vivo* studies revealed an increase in vitamin K, vitamin D and iron delivery, however a variety of methodological discrepancies are apparent in these studies, including the models used, the length of the MNs, the duration of application, temperature control and total micronutrient load in the patches. Data indicate that pre-treating the skin with MNs might enhance delivery; however, a source of variability in the observed effectiveness might include the different molecular weights of the nutrients used, skin factors, the ideal tip radius and MN wall thickness. Non-human studies indicate a potential benefit in combining MN with iontophoresis. Presently, the transdermal delivery seems promising with regard to nutritional supplementation, however limited evidence exists for its efficacy in humans. Future research should aim to control for both intervention duration, possible deficiency status and for the dietary intake of participants.

## 1 Introduction

The evolution of the science of nutrition in parallel to the pharmaceutical industry has led to the development of novel methods for micronutrient delivery. Micronutrient deficiencies currently affect 2 billion of the total world population, and for this, this universal problem is named “hidden hunger” by the World Health Organization [1]. However, although oral nutrient supplements (ONS) might be required, individual characteristics, age and health status particularities, often demand an alternative mode for the delivery of micronutrients. Buccal sprays [2], gums [3], sublingual tabs [4], oral drops, even creams and ointments [5] are often recruited for the delivery of micronutrients, all aiming in enhancing absorption and improving utilization. More recently, the use of adhesive transdermal delivery devices (patches) was suggested for optimal delivery, making use of the body's largest organ, the skin [6].

Within patches, the compounds are stored in a reservoir which is adhesive to the skin on one side, and enclosed with an impermeable backing on the other side [7,8]. The compound is either dissolved in a gel or liquid-based reservoir (allowing for the use of enhancers), or into a solid polymer matrix [7]. The second generation of delivery systems focused on skin permeability enhancement through the use of chemical enhancers (prodrugs, liposomes, microemulsions, etc.) [7,9,10], ultrasound, or iontophoresis. In the latter, charged compounds of small molecules are directed into the stratum corneum via electrophoresis, whereas weakly charged and uncharged compounds are moved by electroosmotic water flow [11]. The third generation of delivery systems includes hypodermic microneedles (MNs) for the enhanced delivery of macromolecules [7].

As with drugs, only a handful of micronutrients are currently delivered via patches [7]. In comparison to the topically applied products, transdermal patches target the systemic circulation of an individual, whereas topically applied compounds target different skin layers, the skin appendages and underlying tissues [12].

## 2 Surpassing the skin barrier

Only drugs with a suitable lipophilicity and a molecular weight <500 Da can be delivered passively through the skin [13]. Moreover, according to some researchers [14,15], to avoid clearance of the particles by macrophages, a size smaller than 500 nm should be sought, with particles smaller than 100 nm tending to move along the edge of the blood stream. Successful compounds delivered via transdermal patches have small molecular masses (some hundred Da), fewer hydrogen bonding sites, a low melting point, require small daily doses, demonstrate moderate lipophilicity, or exhibit octanol-water partition coefficients favoring lipid compounds [7,12,[16], [17], [18]]. With diffusivity being inversely related to the molecular size of the examined compound, the use of large compounds through micron-scale disruptions is most likely to be unsuccessful [19]. Therefore, many compounds do not possess the required physico-chemical characteristics to permeate the skin in adequate quantities, narrowing down the transdermal market [12].

To overcome the skin barrier and reach the intact dermis, alternative pathways mainly for hydrophilic compounds, include blood and lymph vessels, nerve endings, hair follicles and sweat glands [20,21]. Moreover, technologies promoting passive permeation utilize penetration enhancers and as a result, a variety micro and nano-systems have been developed [12,22,23]. On the other hand, active permeation technologies for macronutrient delivery make use of external drivers including electrical (iontophoresis and sonophoresis) [24], and mechanical approaches, with a focus on MN arrays [12,25]. Other existing active delivery technologies like the use of thermal ablation or ultrasound have not yet been examined with regard to micronutrient supplementation [25].

Nevertheless, the topical application of MNs prior to the adhesion of patches consists of a common technique in transdermal patch research, especially in patches lacking MNs themselves. The application of MNs increases the potential of drug delivery through the skin by disrupting the skin layer, creating micro-pathways and leading the compound to the epidermis, thus entering the systemic circulation by surpassing the upper skin layers [26].

## 3 *In vivo* studies

### 3.1 Studies performed on humans

Only two studies to date have tested the micronutrient delivery via transdermal patches on human participants [27,28] (Table 1). However, none of the studies applied a randomized controlled trial (RCT) design. The Saurabh *et al*. study [21] was retrospective and the McCormick [28] one, a non-randomized clinical study. Saurabh and associates [21] examined the efficacy of a transdermal multivitamin (MV) patch against ONS in gastric bypass patients, post-operatively. Their results revealed that participants in the patch group were more likely to demonstrate at least one micronutrient deficiency at 12 months post-operatively, as compared to those receiving ONS in a pill form. In parallel, using the patch for a year was associated with lower serum concentrations of vitamins D, B_1_, and B_12_ [21]. McCormick *et al*. [28] tested the efficacy of iron patches compared to oral iron administration among endurance-trained runners with suboptimal iron stores. In parallel, they [28] were the only ones to record the diet of participants through 4-day food diaries. In the trial, the patch group failed to demonstrate differences in hemoglobin levels post-intervention, whereas at week 6, the *per os* supplementation arm exhibited greater ferritin levels compared to the patch arm participants. In neither of the human studies [27,28] did the patches have MNs, nor was any information on the use of penetration enhancers included in the publications. Moreover, both studies [27,28] used commercially available products from the same company (Patch MD, USA) and this is troubling in extrapolating valid conclusions.

**Table 1:** *In vivo* studies investigating the efficacy of micronutrient supplementation through transdermal patches.

First author	Origin	Sample	Design	Participants	Nutri-ent	Patch/MN details	Intervention	Comparator	Intervention Duration	Results	Diet	Side effects
Saurabh [27]	USA	Humans	Retrospe-ctive	Post-operative LRYGB patients	MV	Patch: MV plus (Patch MD) without MNs	MV patch (*n* = 17)	2 chewable MV, 1 vitamin B_12_ (500 μg), 1 vitamin B complex, 1 Fe (Fe 65 mg or FeSO_4_ 325 mg), 3 Ca with vitamin D (600 mg Ca/800 IU vitamin D) and 1 vitamin D (100 IU) ONS daily (*n* = 27)	12 mo	Vitamin D deficiency was apparent in 81% of the intervention *vs*. 36% of the pill patients. Those in the patch group were more likely to have at least 1 nutrient deficiency. Lower post-operative serum levels of vitamins D, B_1_, and B_12_ were observed in patch participants.	Not accounted for	NR
McCormick [28]	Australia	Humans	Prospective non-RCT	Endurance-trained runners with suboptimal iron stores	Fe	Patch: Iron Plus supplement patch (Patch MD) without MNs	Fe patch (*n* = 14)	Fe pill (*n* = 15)	8 wks	At wk 6, the ONS group had greater Ferritin levels compared with the patch group. There were no differences in Hb pre-intervention to post-intervention in patch.	Diet was monitored (4-d food diary)	None with the patch, 6 with pill (GI issues)
Maurya [30]	India	Rats	Prospective non-RCT	Anemic male Sprague Dawley rats (Charles River, Hollister, CA), weighing 250–275 g	Fe	Patches: Rapidly dissolving MN (with HA), FPP loaded patches	FPP	–	2 wks	Improvement in Hb, RBC, Ht and serum Fe levels.	Controlled diet based on the AIN and a Fe-restricted intake (2–6 ppm)	NR
Modepalli [29]	USA	Rats	Feasibility study	Rats NOD	Fe	Soluble (water soluble polymers) MN array using PMVE/MA	FPP	–	–	Patches dissolved in the skin within in 3–4 h. The recovery of FPP by microdialysis probe in the cutaneous tissue was ~58%. The concentration of free FPP in the dermal interstitial fluid was significant even 10 h after the MN application.	NR	NR
Modepalli [31]	USA	Rats	RCT	Male hairless anemic rats (Charles River, Wilmington, MA)	Fe	Patch: FPP-loaded HPMC gel transdermal patch MN: AdminPen 600 stainless steel MNs (nanoBio-Science LLC. Alameda, CA) for 2′	1) FPP in patches (*n* = 6) 2) FPP in patches with skin pretreated with MN (*n* = 6) 3) FPP in patches with IN (current strength 0.15 mA/cm^2^) (*n* = 6) 4) FPP in patches with IN (current strength 0.15 mA/cm^2^) and skin pretreated with MN (*n* = 6)	1) placebo patch (HPMC gel patch without FPP) (*n* = 6) 2) IP FPP administration (*n* = 6)	4 wks	No improvement was noted in the hematologic parameters of the placebo and passive FPP patch delivery (Groups 1 and 3). No improvement was noted in the hematologic parameters or morphology of RBC in groups 2 & 4, indicating that the amount of delivered FPP was suboptimal.	Regular, standard diet	NR
Murthy [32] 2009	USA	Rats	RCT	Male hairless rats (Charles River, Wilmington, MA)	Fe	Patch: A polyethylene chamber of 1 cm^2^ area glued on the skin surface with cyanoacrylate glue (Krazy Glue, Elmers Products Inc., Columbus, OH) and FPP in the chamber	1) FPP on skin surface chambers (*n* = 3) 2) FPP on skin surface chambers with IN (current of 0.5 mA/cm^2^) (*n* = 3)	IV FPP delivery via tail vein injection (*n* = 3)	6 h	In the 1st group, serum Fe and %TS did not change significantly. In the IN group, total serum Fe and %TS increased at 3 h and remained TA, even after 12 h.	NR	Mild skin redness which disappeared within 5–6 h
Juluri [33]	USA	Rats	RCT	Male hairless anemic rats (Charles River, Wilmington, MA), 8 wks old, weighing 250–300 g	Fe	Patch: transdermal Polyolefin foam patch loaded with 200 μL of 50 mg/mL ID, placed on the dorsal side (6 h)	1) ID patch (*n* = 6) 2) ID patch with skin pretreated with MN (2′) (*n* = 6)	ID via IP delivery (*n* = 6)	3 wks	No improvement in the hematological parameters in the ID patch group, whereas, in case of MN pretreated and IP group, an improvement was observed at 2–3 wks.	Low-Fe diet	Skin discoloring after MN treatment
Yamagishi [34]	Japan	Dairy cattle	Prospective non-RCT	Healthy, non-pregnant Jersey heifers	Ca	Patch: reservoir-type transdermal patch	1) CAL (*n* = 2) 2) CAL and C_12_H_27_N (*n* = 2)	Control vehicle (*n* = 2)	3 wks	Cattle receiving CAL or CAL + C_12_H_27_N had greater increases in plasma CAL and Ca levels on days 2 and 3. The plasma AUC for CAL and Ca in the CAL and CAL + C_12_H_27_N arms increased compared to the controls.	NR	NR

AIN: American Institute of Nutrition; AUC: Areas under the curve; C_12_H_27_N: dodecylamine; Ca: Calcium; CAL: calcitriol; Fe: Iron; FeSO4: Ferrous sulfate; FPP: ferric pyrophosphate; GI: gastrointestinal; HA: hyaluronic acid; Hb: Hemoglobin; Ht: Hematocrit; HPMC: Hydroxypropyl methyl cellulose; ID: Iron dextran; IN: iontophoresis at a current strength of 0.15 mA/cm^2^ strength; IP: Intraperitoneal; LRYGB: Laparoscopic Roux-en-Y gastric bypass; MN: microneedle; MV: multivitamin; NOD: Not other defined; NR: not reported; ONS: Oral Nutrient Supplement; PMVE/MA: poly methylvinylether/maelic acid; RBC: Red Blood Cell; RCT: randomized controlled trial; TS: Transferrin saturation.

### 3.2 Animal studies

Rats were used in the majority of animal *in vivo* studies [[29], [30], [31], [32], [33]], whereas in one study, dairy cattle [34] were employed (Table 1). Most *in vivo* animal studies had a non-randomized prospective design [29,30,34], and few were RCTs [[31], [32], [33]]. All *in vivo* studies performed on rats evaluated the delivery of iron, using either anemic hairless rats [[30], [31], [32], [33]], or rats without further specifications [29]. In these, iron was either delivered in the form of ferric pyrophosphate (FPP) or as iron dextran (ID) through reservoir-type patches [32], simple gel/foam patches without MN [31,33], patches with dissolving MNs [29,30], or patches with stainless steel MNs [31]. Among those using patches without MNs [31,33], in two studies the skin of the participating animals was pretreated with MNs to increase permeability [31,33]. Iontophoresis (IN) was applied in two studies [31,32], using a current strength of 0.15–0.5 mA/cm^2^. Intraperitoneal and intravenous (IV) administration was used as a positive control method in one [33] and two studies [31,32], respectively.

Animals were either fed a regular standard diet [31], a low iron diet [33], a controlled low iron diet otherwise based on the American Institute of Nutrition guidelines [30], or, the dietary intake of the animals was not accounted for [27,29,32,34].

In one prospective non-RCT, healthy, non-pregnant Jersey heifers were used as a population [34] and calcium was the administered nutrient, in the form of calcitriol or calcitriol with concomitant dodecylamine delivery, using fabricated transdermal reservoir-type patches and without controlling for the cattle's diet.

With regard to the delivery of iron, Modepalli and associates [29] failed to record changes in the hematological parameters of the sample, as their study was mostly a feasibility one. In studies where the applied patches did not incorporate MNs, the results did not appear to different significantly from the baseline [[31], [32], [33]]. When patches with dissolving MNs were employed [30], improvements were noted in the Hb, RBC, Ht and serum Fe levels [30] of the participating rats.

On the other hand, when rat skin was pretreated with MNs [31,33], ambiguous results were noted. Modepalli and associates [31] failed to induce a significant improvement in either the hematological parameters or the morphology of RBC of the participating rats, suggesting that possibly, the amount of administered FPP was suboptimal. On the other hand, Juluri [33] reported improved hematological parameters 2–3 weeks post-trial initiation. However, in the first [31], rats were fed a regular diet, whereas in the second [33], a low-iron diet. Thus, it is possible that in the Modepalli trial [31], dietary iron intake might have compromised the induced efficacy of the intervention, whereas on the other hand, in the Juluri intervention [33], the constant low dietary iron intake might have allowed for a greater hematological improvement during the study.

When IN was applied [31,32] in the interventions, an acute increase in serum Fe and % transferrin saturation (TS) levels was noted when FPP was delivered via skin surface chambers, with IN at a current of 0.5 mA/cm^2^ [32]. When the duration of FPP patches application lasted for 4 weeks in total with concurrent IN at a constant current strength of 0.15 mA/cm^2^ and the skin was pretreated with MNs [31], no significant improvements were recorded in the hematologic parameters of participating rats. However, in the latter study [31], the intervention was long-term, the current strength was much lower as compared to the first study, and rats were fed a regular, uncontrolled diet.

In the calcium-intervention trial, Yamagishi and associates [34] demonstrated increased serum calcium levels in both groups receiving either calcitriol, or calcitriol with concomitant dodecylamine intake, through fabricated reservoir-type patches, as compared to control vehicles. The rise was noted on the 3rd day of delivery initiation and remained similar throughout the 3-week trial.

### 3.3 Adverse events of *in vivo* studies

Among the studies using human populations, McCormick [28] reported few gastro-intestinal adverse events associated with the pill administration and none in the patch arm. By design, physiological adverse events could not be reported in animal studies, however, a mild redness of the skin was observed in rats receiving FPP through patches [32], as well as a skin decolorization among rats being pre-treated with MNs before an ID patch was applied transdermally [33].

## 4 *Ex vivo* and *in vitro* studies

*In vitro* [29,33,35] and *ex vivo* [14,30,31] studies examining the transdermal delivery of micronutrients through patches are described in Table 2. All studies utilized porcine [14,35], or rat [30,31,33] skin models, with the exception of Modepalli [29], who used human models. Although Park and associates [24] also tested the transdermal delivery of micronutrients (retinol, niacin, and glutamic acid) using the dorsal skin of mini-pigs *in vitro*, the aim of their study was not dietary supplementation, but cosmetic. Subsequently, that study was not considered as relevant to the present review.

**Table 2:** *Ex vivo* and *in vitro* studies assessing the efficacy and permeability of micronutrient delivery transdermal patches.

First author	Origin	Study type	Tested nutrient	Micronutrient delivery form	Delivery method	Samples	Control	Temperature control	Results	Issues
Juluri [33]	USA	*in vitro*	Iron	ID	ID patch (transdermal Polyolefin foam patch loaded with 200 μL of 50 mg/mL ID) with skin pretreated with MNs (2′)	Male hairless rat skin (Charles River, Wilmington, MA)	ID via IP delivery	✓	The cumulative amount of ID permeated at the end of 6 h was 10.28 ± 0.45 μg/cm^2^. After 6 h of permeation 2.48 μg/mg of ID was retained in the skin.	
Hutton [35]	UK	*in vitro*	Vitamin K	Vitamin K	Vitamin K MN dissolving arrays (using an aqueous blend of Gantrez® S-97 and Tween® 80)	Neonatal porcine skin	–	✓	Permeation of vitamin K through porcine skin occurred throughout the 24 h experiment, with MN arrays delivering 1.80 ± 0.08 mg of vitamin K during this time (35% of the administered dose).	Small study duration. Did not measure the time needed to dissolve MN arrays.
Kim [14]	S. Korea	*ex vivo*	Vitamin D_3_	PLGA nanoparticles loaded with Vitamin D_3_ and PVA stabilizer	Coated MN patch	Porcine skin (Cronex, Hwasung, South Korea)	Transdermal cream with identical vitamin D_3_ amounts and a penetration enhancer [45]	–	Despite the fact that the transdermal cream contained a chemical penetration enhancer, the MN system showed 5-fold better delivery performance.	Is the encapsulation capacity able to carry daily human needs? 25 μg were used
Maurya [30]	India	*ex vivo*	Iron	FPP loaded HA	MN (polydimethylsiloxane micromold) patch	Excised rat skin	–	–	The mean Fe recovered from the skin after 5′ application of the patch was 130.5 ± 18.6 mg (66% of the MN total load).	
Modepalli [31]	USA	*ex vivo*	Iron	1) FPP patches 2) FPP patches with skin pretreated with MN injection 3) FPP patches + IN 4) FPP patches + IN, with skin pretreated with MN injection	Patch: FPP loaded HPMC transdermal patch MN: AdminPen 600 stainless steel with an area of 1 cm^2^ and 187 MNs with a height of 500 μm (nanoBioScience LLC. Alameda, CA) applied for 2′	Excised rat abdominal skin	–	✓	The lowest amount of FPP was permeated at patches alone, followed by patches with IN, MN pretreated patches and finally the MN + IN pretreated patches induced the greatest (~ 44-fold) enhancement in the flux (51.24 ± 7.55 μg/cm^2^/h) over passive permeation.	
Modepalli [29]	USA	*in vitro*	Iron	FPP in soluble MN arrays	Soluble (15% w/w PMVE/MA) MN arrays patch	Human HDF [CCD1093Sk (ATCC® CRL2115™)] cell lines (ATCC, Manassas, VA)	–	–	Based on the safety and toxicity study, the amount of FPP in the patches was safe and non-toxic.	Feasibility study

FPP: ferric pyrophosphate; HA: hyaluronic acid; HDF: Human Skin Fibroblast; HPMC: Hydroxypropyl methyl cellulose; ID: iron-dextran; IN: iontophoresis at a current strength of 0.15 mA/cm^2^; MN: Microneedle; MTS: mitochondrial activity; PLGA: Poly (lactic-co-glycolic acid); PMVE/MA: poly methylvinylether/maleic acid; PVA: polyvinyl alcohol.

Vitamin K [35], vitamin D_3_ [14] and iron [[29], [30], [31],^33^], in the form of ID or FPP were the delivered micronutrients using patches [31,33], patches on skin pretreated with MNs [31,33], dissolving MNs [29,35], MNs coated with the micronutrient [14], or micromold MNs [30]. IN was applied in one study [31] only (delivering FPP). Some studies [31,33,35] reported retaining constant temperature throughout the patch application to minimize permeation differences, however, others did not report any temperature control [14,30].

According to Juluri and associates [33], MN pre-treatment leads to the delivery of a substantial amount of ID across the skin and the colloidal ID does not appear to penetrate or permeate across the intact skin in detectable amounts.

A variety of methodological differences are apparent in the published research using animal models, including the washing of skin with water prior to the treatment, as well as the methods used to assess the dissolution of MNs. Moreover, length (height) of MNs varied greatly, ranging from 467.59 ± 15.23 mm [30] to 700 μm [14]. Patch-application duration was also different in most research, with Hutton and associates [33] reporting 24 h (30 s of constant finger pressure and a 5.0 g circular stainless steel weight then placed on top), Modepalli [29] and Juluri [33] applying the patch for 2 min in total, Maurya [30] and Kim [14] reporting a 5 min application, and Modepalli [29] applying the patch on the model for 1 h. Thus, depending on the model used, the duration and mode of the application and the height of the MNs, differences were also observed in the reported micronutrient load in the skin post-application, ranging from 35% of the total MN initial load [35] to 81.08% [14].

## 5 To microneedle or not?

In most *in vivo* studies with the exception of those performed on humans and cattle [27,28,34], either the patches applied contained MNs, or the skin of the subjects was pre-treated with MNs. In the human studies [27,28], iron was the delivered micronutrient of interest with both studies revealing a greater improvement in the ONS arm as compared to the patch-receiving participants. Although none reported the form of iron used in the patches (both used a commercially available product), in the case of FPP which is the most common form of iron used, the molecular weight reached 745 Da, which might in part explain the poor permeation of Iron [31]. Μoreover, as already explained, none of the human studies used MN technology, which might have increased micronutrient delivery.

When FPP patches with MNs were applied in rats without a comparator arm [29,30], improvements were noted in hematological (Hb, RBC, Ht and Fe levels) parameters [30] and the concentration of free-FPP in the dermal interstitial fluid [29] as compared to the baseline. When patches with MNs or skin pre-treated with MNs were compared against patches without MN [31,33], penetration and delivery of FPP/ID was enhanced in the first groups, as compared to the latter. Similar findings were also reported in *ex vivo* experiments [31], with MN pretreated skin inducing a greatest enhancement in FPP uptake by the skin as compared to the application of passive patches alone. Researchers [30,31] also noted that poor delivery might also be the result of MNs penetrating only the upper layers of the skin, given that the length of MNs is short enough to avoid possible stimulation of pain receptors [36].

In the case of calcitriol delivery via patches without MNs [34], an improvement was noted in the cattle with regard to plasma calcitriol and Ca concentrations compared to the controls (no calcitriol delivery), however, the molecular weight of calcitriol is 416.64 Da, thus, a greater passive permeation is expected as compared to the FPP.

With regard to the delivery of vitamin D_3_, coated MN patches induced improved delivery performance (5-fold) *ex vivo*, as compared to ointment with a similar vitamin D_3_ content [14]. According to an *in vitro* study of vitamin K delivery without a comparator arm, delivery of vitamin K with MN patches was optimal, reaching 35% of the administered dose [35]. Nevertheless, all *in vitro* and *ex vivo* studies lack the assessment of hematological parameters which would either prove, or refute the clinical efficacy of the intervention.

MNs have been suggested to deliver a variety of compounds in a less invasive and painless manner as compared to the hypodermic needles [37], while their composition may vary greatly. Moreover, differences also arise depending on the use of patches with MNs, or pretreating the skin with MNs prior to the application of patches. Although this issue was not addressed in any of the studies reviewed herein, we are unsure of which method is more efficient in drug delivery, while carrying the fewer adverse events. Often, when polymers are used to create MNs, possible discharge into the skin is another issue of concern, which can be surpassed with the use of biodegradable polymeric MNs [37]. Other issues regarding the use of MNs include the optimal ratio of MN fracture force/skin insertion force, the ideal tip radius and MN wall thickness required to induce an improved delivery [38]. In this manner, great variability is observed in all studies reported herein, with many researchers failing to report relevant and immediately comparable data.

## 6 Effects of iontophoresis

To further enhance the transdermal delivery of FPP, iontophoresis has been suggested as a complementary practice, provided that the stratus corneum is compromised [31]. Nanoparticles with a negative charge have been considered as more efficientin entering the blood circulation and avoiding the phagocytic procedures [14,38].

*In vitro* experiments, indicated that cathodal iontophoresis in MN-pre-treated skin enhanced the delivery of FPP considerably, as compared to MNs alone, or passive transdermal delivery [31]. *In vivo* animal experiments comparing iontophoresis to MNs, reported a lack of significant improvement regarding the hematologic parameters and morphology of RBC, indicating that possibly the amount of FPP delivered was suboptimal [31]. As compared to passive transdermal delivery [32], iontophoresis induced acute improvements in the total serum Fe and TS, within hours of the patch application.

Moreover, a combination of MN pretreatment with iontophoresis resulted in significant improvements concerning the hematologic and biochemical parameters of rats (RBC, MCV, MCH and MCHC), within four weeks of intervention in anemic rats [31]. Similar observations were also reported in *ex vivo* experiments [31], with a combination of MNs application and iontophoresis producing the greatest enhancement (44-fold) in the FPP skin uptake.

## 7 Research in the pipeline

At the moment, three studies testing the delivery of micronutrients via transdermal patches on humans are in the pipeline (Table 3). Two of these are being conducted in the USA using patch MD products and one is being conducted in Ireland. Of these, one is aiming in reducing deficiencies in bariatric surgery patients and the rest are using general population samples, testing the efficacy of transdermal patches compared to patches of smaller surface, or other modes of micronutrient delivery (chewable, quick dissolve ONS, etc.) in comparative effectiveness designs.

**Table 3:** Research pipeline.

Registration number	Origin	Institute	Design	Study Duration	Participants	Intervention	Comparator	Duration	Outcomes
**NCT03360435**^a^	USA	University of Florida	Observational cohort	Dec 2015–Sep 2021	N = 100 adult bariatric surgery post-operative patients	MV transdermal patch containing vitamins A, D, E, K_2_, C, thiamine, riboflavin, niacin, pantothenic acid, pyridoxine, biotin, folic acid, Ca, Fe, P, I, Mg, Zn, Se, Cu, Mn, Cr, Mb, K, Cl, B and cyanocobalamin	N/A	1 yr	Percentage of subjects with deficiencies, constipation, diarrhea, indigestion, acid reflux, abdominal pain, PTH, Fe, Cu, Ferritin, thiamin, TIBC, pyridoxine, Zn, Ca, methylcobalamin, vitamin D, folate
**NCT04851990**^a^	Ireland	Atlantia Food Clinical Trials	Parallel arm RCT	NR	N = 30 adults	large patch containing vitamin D (30,000 IU) & dextrose (13 mg) applied daily	small patch with vitamin D (30,000 IU) & dextrose (13 mg) applied daily	8 wks	number of participants with TEAE, discontinuation due to TEAE, blood safety parameters (full blood count, FPG, TC, HDL, LDL, TG, bilirubin, Ca, protein, albumin, globulin, creatinine, urea, uric acid, Na, K, Cl, bicarbonate, Mg, PO₄³^-^, 25(OH)D), liver function (ALT, ALP, AST, GGT), blood pressure, heart rate, body temperature
**NCT02686905**^a^	USA	California State Polytechnic University	Parallel arm^b^ RCT	Feb 2016–NR	N = 30 adults	1) MV patch 2) Vitamin D_3_ + Ca patch 3) Vitamin B_12_ patch	1) Chewable MV with Fe 2) Chewable Ca 3) Quick dissolve vitamin B_12_	3 mo	FPG, Ca, Ferritin, B_12_, vitamin D, HbA1c, fat mass, body weight, waist and hips circumferences, stature, TBW

ALP: alkaline phosphatase; ALT: alanine aminotransferase; AST: aspartate aminotransferase; FPG: fasting plasma glucose; GGT: gama-glutamyl transferase; HDL: high-density lipoprotein; LDL: low-density lipoprotein; MV: multivitamin; N/A: Not applicable; NR: not reported; PO₄³^-^: Phosphate; PTH: Parathyroid hormone; RCT: Randomized controlled trial; TEAE: treatment-emergent adverse event; TBW: total body water; TC: total cholesterol; TG: triglycerides; TIBC: total iron binding capacity.

a Active but with unknown status.

b Given the small number of participants and the plethora of interventions and comparators, a cross-over design is more likely to occur.

Unfortunately, the D3forME trial (NCT02174718), was discontinued due to manufacturing issues regarding the patch used.

## 8 Overview of the advantages of using the transdermal patches technology

**Fig. 1:**
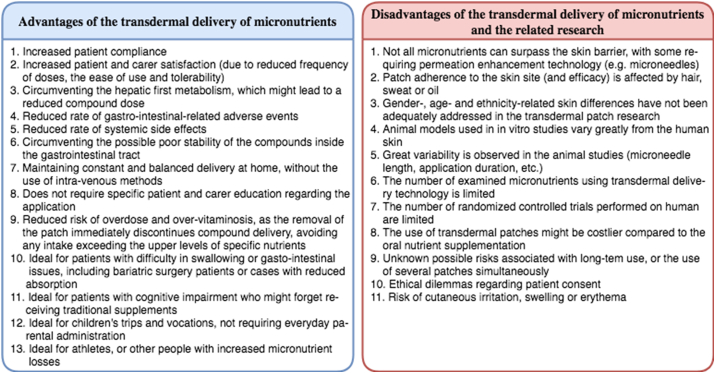
Possible advantages and disadvantages/risks associated with the transdermal delivery of micronutrients and research gaps.

According to Isaac and Holvey [39], the delivery of drugs via transdermal patches entails a variety advantages for the patient (Fig. 1), encouraging patient compliance [40]. First, the use of patches is associated with increased patient and carer satisfaction, due to the reduced frequency of doses, the ease of use and tolerability (depending on the adverse events) [21,39,41]. When children are the patients using the patches, the transdermal technology offers the opportunity to surpass the often unpleasant and inconvenient parental administration associated with ONS [39]. In parallel, the delivery of drugs/compounds through patches is circumventing the hepatic first metabolism, which might lead to a reduced compound dose as compared to a *per os* administration [39,42]. In the traditional ONS administration, an increased rate of gastro-intestinal-related adverse events is noted and often, poor stability of the compounds inside the gastrointestinal tract is also apparent [43]. On the other hand, the use of transdermal delivery technology is associated with reduced systemic side effects [44]. In terms of maintaining a constant balanced delivery, patches appear superior compared to the *per os* administration, in maintaining constant blood levels instead of episodic peaks [39]. As compared to the IV delivery, patches can be administered and used outside the hospital setting, by patients themselves, or by their carers. Patches are ideal for the delivery of micronutrients in patients with swallowing difficulties, or gastro-intestinal issues, as well as for those with cognitive impairment, likely to miss doses of traditional ONS. Moreover, they also form a good solution for children, travel and vocation, which typically requires everyday parental administration in ONS form.

Finally, patches can potentially reduce the risk of overdose and over-vitaminosis, as the removal of the patch immediately discontinues compound delivery, avoiding any intake exceeding the upper levels of specific nutrients [39].

## 9 Limitations of the transdermal delivery of micronutrients and the related-research

Limitations of the transdermal delivery (Fig. 1) include the difficulty to surpass the skin barriers, especially when lipophilic compounds are concerned, like vitamin D [45]. On the other hand, as compared to topical solutions or passive delivery, patches using MN appear to deliver a greater amount of micronutrient to the epidermis or upper dermis region, and from there into the circulation [26]. As a result, the transdermal patches technology is associated with slower time towards peak blood concentrations, thus, this model is not suited for emergency treatments requiring the rapid release of nutrients and a fast peak in blood concentrations [39].

The prerequisites for the improved bioavailability of nutrient have already been discussed (molecular weight, few hydrogen bonding sites, low melting point, moderate lipophilicity, etc.) [7,12,[16], [17], [18]]. These cumulatively limit the choices of nutrients that can be delivered using the transdermal format [39].

Moreover, according to Isaac and Halvey [39], good patch adherence to the skin is required for the increased effectiveness of patches. The presence of sweat, scars, hair or oil, on the application site might reduce adherence and limit absorption [39]. Thus, specific guidance must be provided in the commercial patches packages in order to guide proper use and increase effectiveness. Moreover, research on the transdermal delivery of micronutrients has not yet assessed variations in the delivery efficacy as a result of inadequate patch adherence, nor has a specific application site been identified as more effective.

Gender differences exist in the human skin, including the keratinocyte size, with male skin samples tending to be larger than those from obtained from female donors [46]. In parallel, men have larger skin pores sizes, more active sebaceous glands and a lower skin pH compared with the women [[46], [47], [48]]. Moreover, ethnicity- and age-based differences are also apparent, with Afro-Caribbean skin demonstrating a reeduced permeation compared to the Caucasian [49,50], and younger skin exhibiting increased permeability in contrast to the older one, possibly increasing the efficacy of transdermal therapy [46]. Thus, it appears that one size does not fit all and it is possible that the application of the same patch might induce different efficacy on different subjects.

An additional limitation is the nature of animal studies as the majority of *in vitro* studies employ rat models, given that the use of human skin is costly and raises a variety of ethical concerns [51]. According to van Ravenzwaay and Leibold [52], rat skin dermal penetration *in vitro* is higher than *in vivo*. In parallel, rat skin is more permeable to all substances as compared to the human skin [52]. On the other hand, based on the literature [53], mean thickness of rat skin is much lower to that of humans and great inter-individual variation has also been reported in human skin samples, depending on the age, body site, and skin type, pigmentation, gender, blood content, and lifestyle [54]. Moreover, the metabolic, surveillance, and transport processes taking place in the deep skin layers can also alter permeability and efficacy of transdermal products [55]. Therefore, the efficacy of MNs depends greatly on the diverse mechanical properties of the skin between the species [53]. Given that quite often researchers noted differences in the permeability of animal skin, translating the possible efficacy of animal studies for human use consists of findings extrapolation [53].

Transdermal delivery via ointments and creams also carries a variety of bottlenecks (depending on the nutrients used), and has been criticized [56]. In parallel, it is challenging as each nutrient has a different molecular weight, and side effects, when used topically.

Moreover, it has been argued that the use of transdermal delivery devices for micronutrients might be costlier compared to the traditional ONS [39], although no research has evaluated this yet. Questions have also been raised with regard to the cumulative effects of long-term use and the possible risks associated with the use of multiple patches simultaneously [39]. As seen in animal studies, the use of transdermal devices often triggers skin allergic reactions, cutaneous irritation, erythema or swelling [44], although this has not been verified on humans.

Finally, ethical and legal dilemmas are apparent in cases when consent to treatment cannot be provided by the patient [39], or when the patient refuses to consume traditional ONS.

## 10 Conclusions

The transdermal delivery of micronutrients is an ambitious domain in clinical research with important ramifications for public health. Postulated advantages of delivering micronutrients transdermally include avoiding the first-pass effect of the liver, reducing gastrointestinal related side-effects and providing a stable release rate for a longer time [7]. In parallel, transdermal delivery provides a highly convenient and pain-free administration platform for patients [44], limiting non-compliance associated with pain, swallowing, age or other individual particularities. Subsequently, patient acceptability of all transdermal products appears high [44].

Apart from enhanced skin penetration, continued evolution of the drug industry for topical and transdermal delivery focuses on novel technologies controlling doses, site-targeted delivery, multiplying the range of compounds that can be delivered via skin patches [12]. On the other hand, novel systems including pharmaceutical jewelry [57] have been incorporated in the transdermal delivery science and are expected to expand their application in the micronutrient market as well.

The present review indicates the limited number of studies conducted on humans and the variability in the design and methodology observed in animal research. Thus, it appears that research is still in premature stages and although promising and important, we cannot yet conclude on the efficacy of the transdermal micronutrient delivery on humans.

## Consent to publish

Not applicable.

## Availability of data and materials

Not applicable as this is a review study.

## Funding

No funding was obtained for the present study.

## Authors’ contributions

All authors were involved in drafting the manuscript, read and approved the final draft of the manuscript.

## Declaration of competing interest

All the authors declare that there is no conflict of interest.
